# Predictive Factors of Early Response to Dupilumab in Patients with Moderate-to-Severe Atopic Dermatitis

**DOI:** 10.3390/jcm12206575

**Published:** 2023-10-17

**Authors:** Silvia Ferrucci, Giovanni Casazza, Martina Zussino, Simona Tavecchio, Angelo V. Marzano, Micol Tedeschi

**Affiliations:** 1 Dermatology Unit, Fondazione IRCCS Ca’ Granda Ospedale Maggiore Policlinico, 20122 Milan, Italy; silvia.ferrucci@policlinico.mi.it (S.F.); martina.zussino@policlinico.mi.it (M.Z.); angelo.marzano@unimi.it (A.V.M.); 2 Department of Clinical Sciences and Community Health, Università Degli Studi di Milano, 20122 Milan, Italy; giovanni.casazza@unimi.it; 3 Department of Pathophysiology and Transplantation, Università Degli Studi di Milano, 20122 Milan, Italy; simona.tavecchio@gmail.com; 4 Pediatric Unit, Department of Clinical, Surgical, Diagnostic and Pediatric Sciences, University of Pavia, 27100 Pavia, Italy

**Keywords:** atopic dermatitis, dupilumab, early reposnders

## Abstract

Efforts have been made to identify factors influencing clinical response in patients with atopic dermatitis (AD) treated with dupilumab. A retrospective single-center observational study was carried out by analyzing data from 492 patients aged 12 years and older with moderate-to-severe AD. The study aimed to identify baseline demographic and clinical factors that could predict the achievement of a mild level of disease, i.e., an Eczema Area and Severity Index (EASI) ≤ 7, within 4 weeks from dupilumab initiation. Classic, generalized lichenoid and inflammatory phenotypes compared with a nummular eczema phenotype (OR = 6.9, 95% CI 2.04–23.48 and OR = 4.22, 95% CI 1.22–14.66, respectively) and a baseline EASI ≤ 24 and between 24–29, compared with a baseline EASI ≥ 29 (OR = 3.1, 95% CI 1.81–5.41 and OR = 1.8, 95% CI 1.05–3.07, respectively), were found to be predictive factors of early response to dupilumab, highlighting the importance of early biological treatment of AD.

## 1. Introduction

Atopic dermatitis (AD) is a multifactorial, chronic inflammatory skin disorder that predominantly affects infants and children (20%), with a 2–8% prevalence in the adult population [1], imparting a substantial burden on affected individuals and healthcare systems worldwide [2].

The pathophysiology of AD is the result of a complex interplay between genetic predisposition, immunological triggers, and environmental triggers, making it a subject of significant research interest within the field of dermatology and clinical immunology [3].

Individuals with a family history of AD, asthma, or allergic rhinitis are at an increased risk of developing the condition, highlighting the genetic component of AD susceptibility. Furthermore, mutations in genes associated with skin barrier function, such as filaggrin, keratinocyte differentiation, and cornified envelope formation, have been implicated in the pathogenesis of AD, underscoring the importance of the skin barrier in disease development [4,5]. Disruption of the epidermal barrier allows for increased transepidermal water loss and the penetration of allergens and irritants, exacerbating inflammation and leading to further skin damage [6].

Immunologically, AD is characterized by dysregulated immune responses, with a predominance of T-helper 2 (Th2) cell-driven inflammation. Th2 cytokines, such as interleukin-4 (IL-4) and interleukin-13 (IL-13), play a major role in orchestrating the inflammatory cascade, contributing to the hallmark features of AD both in the acute and chronic phases, including pruritus and eczematous skin lesions [7,8].

The management of AD is multifaceted and often requires a combination of therapeutic approaches. Treatment options range from topical emollients, corticosteroids, and immunomodulatory agents, including topical calcineurin inhibitors, to more advanced systemic agents for patients affected by moderate-to-severe AD, who have experienced inadequate relief from topical therapies [9].

Dupilumab is the first biologic therapy approved for moderate-to-severe AD in adults, adolescents, children, and infants. Dupilumab is a fully human IgG4 monoclonal antibody that exerts its therapeutic effects by binding to the common α-subunit of IL-4 and IL-13 receptors, playing a pivotal role in orchestrating the Th2 inflammatory cascade [10]. It has demonstrated significant efficacy in reducing disease severity, alleviating pruritus, and improving quality of life as assessed in the SOLO 1 and 2 16-week-studies [11], in the AD CAFÉ trial [12] with a concurrent administration of the drug alongside topical corticosteroids, and in the 52-week CHRONOS trial [13]. The approval of dupilumab as a monotherapy, in conjunction with topical corticosteroids, for a wide age range including adolescents, children, toddlers, and infants, was grounded on the outcomes of randomized, double-blind, placebo-controlled clinical trials: LIBERTY-AD-ADOL [14] for adolescents, LIBERTY AD PEDS [15] for children aged 6 to 11 years, and LIBERTY AD PRESCHOOL [16] for the very young, ranging from 6 months to 5 years of age. The clinical effectiveness of these latest clinical trials was subsequently confirmed in real-world samples of children and adolescents aged 6 to 11 years [17] and over 12 years [18]. Moreover, its safety profile has made it a valuable addition to the therapeutic armamentarium for AD, ushering in a new era of precision medicine [19].

Recently, efforts have been made to identify possible predictors of clinical response to dupilumab in terms of improvement of disease severity, symptoms, and quality of life, but so far, no conclusive data have been obtained [20].

### Learning Points

- Dupilumab is an effective and safe treatment for patients affected by moderate-to-severe AD.
- Our findings demonstrate that patients affected by classic, generalized lichenoid and inflammatory phenotypes reach a mild level of disease earlier than patients with other non-classic phenotypes of AD.
- Patients with an EASI < 29 and particularly ≤24 respond earlier to dupilumab compared with those who have severe dermatitis with an EASI ≥ 29.
- Total serum IgE levels and eosinophil count values at baseline were not associated with an early clinical response.
- An association was found between presence of atopic blepharoconjunctivitis and facial involvement at baseline with the development, respectively, of blepharoconjunctivitis and facial redness dermatitis as adverse events.
- A timely administration of dupilumab, when EASI is <29, can effectively and rapidly bring the disease under control.

## 2. Methods

### 2.1. Study Design

A retrospective, single-center observational study was carried out in patients diagnosed with moderate-to-severe AD to identify predictors of an early response to dupilumab therapy, defined by the achievement of a mild disease state.

The analysis encompassed clinical data from 492 patients, comprising both adults and adolescents aged over 12 years, collected between June 2018 and March 2022 at the Dermatology Unit of Fondazione IRCCS Ca’ Granda Ospedale Maggiore Policlinico in Milan. All patients included in the study were affected by moderate-to-severe AD and treated with dupilumab in accordance with the recommendations and reimbursement guidelines set forth by the Italian Drug Agency (AIFA) [21]. In Italy, reimbursement for dupilumab is available to adults with moderate-to-severe AD, as indicated by an Eczema Area and Severity Index (EASI) [22] score ≥ 24, who have demonstrated inadequate responses to topical therapies or have not experienced a satisfactory response to or tolerated cyclosporine, or for whom cyclosporine is contraindicated. For adolescents, reimbursement is also applicable in cases of moderate AD with an EASI score ≥ 16, provided they exhibit at least one of the following criteria: an Itch Numerical Rating Scale (itch-NRS [23]) score ≥ 7, a Dermatology Quality of Life Index (DLQI [24]) score ≥ 10, or the involvement of sensitive areas such as the face, hands, or genitalia.

The treatment regimen involved an initial subcutaneous loading dose of 600 mg administered via prefilled syringe at the baseline, followed by 300 mg every other week. For patients weighing fewer than 60 kg, the loading dose was adjusted to 400 mg, followed by 200 mg once every two weeks [21]. Throughout the study, patients were permitted to continue any approved topical AD treatments and moisturizers as needed.

All procedures adhered to the Declaration of Helsinki of 1964 (revised in 2013). The study protocol was approved by the local review board. All patients granted written informed consent to the investigators for data extraction from patient records.

Clinical response was evaluated in terms of the severity and extent of dermatitis, itching, sleep disturbances, and quality of life. The severity and extent of AD were quantified utilizing the EASI score [22], which considers erythema, infiltration, excoriation, and lichenification as its key parameters. An EASI score range from 0 to 72, with higher values, was indicative of increased disease severity. Furthermore, pruritus, sleep disturbances, and the impact on quality of life were evaluated using the itch-NRS [23], which rates peak pruritus experienced over the past 7 days on a scale of 0 to 10 (with 10 representing the most severe itching imaginable); the sleep-NRS [25] for the past 7 days (ranging from 0 to 10, reflecting greater sleep disturbances); and the DLQI [24], which ranges from 0 to 30, with higher scores indicating a more pronounced adverse impact on overall quality of life.

The primary objective of this investigation was to discern the patient-specific baseline characteristics exerting influence on treatment outcomes at 4 weeks (T1) as well as the subsequent assessments at 16 weeks (T4), 32 weeks (T8), and 52 weeks (T12), with specific emphasis on the early treatment response at T1.

The criteria for treatment early-response were defined as achieving a mild level of disease, characterized by an EASI score of ≤7, within the first 4 weeks following the initiation of therapy [26]. Within the study context, patients were categorized into different groups based on their response timing to the treatment. Specifically, those who showed improvement in their clinical condition by achieving an EASI score of ≤7 within the initial 16 weeks (T4) were categorized as “intermediate responders”. On the other hand, “late responders” were identified among patients who exhibited clinical improvement in terms of disease severity at T8 or T12, corresponding to the time frame between 32 and 52 weeks from the treatment’s commencement. Individuals who did not attain disease remission by T12 were classified as non-responders, or, if they displayed clinical improvement that was insufficient to qualify as responders but still fell short of the criteria for moderate-to-severe disease, they were categorized as partial responders, with an EASI score ranging from 7 to 16 (7 < EASI ≤ 16). This categorization was employed to facilitate a comparison of patients’ varying response times to dupilumab treatment within the study’s scope.

**Figure 1:**
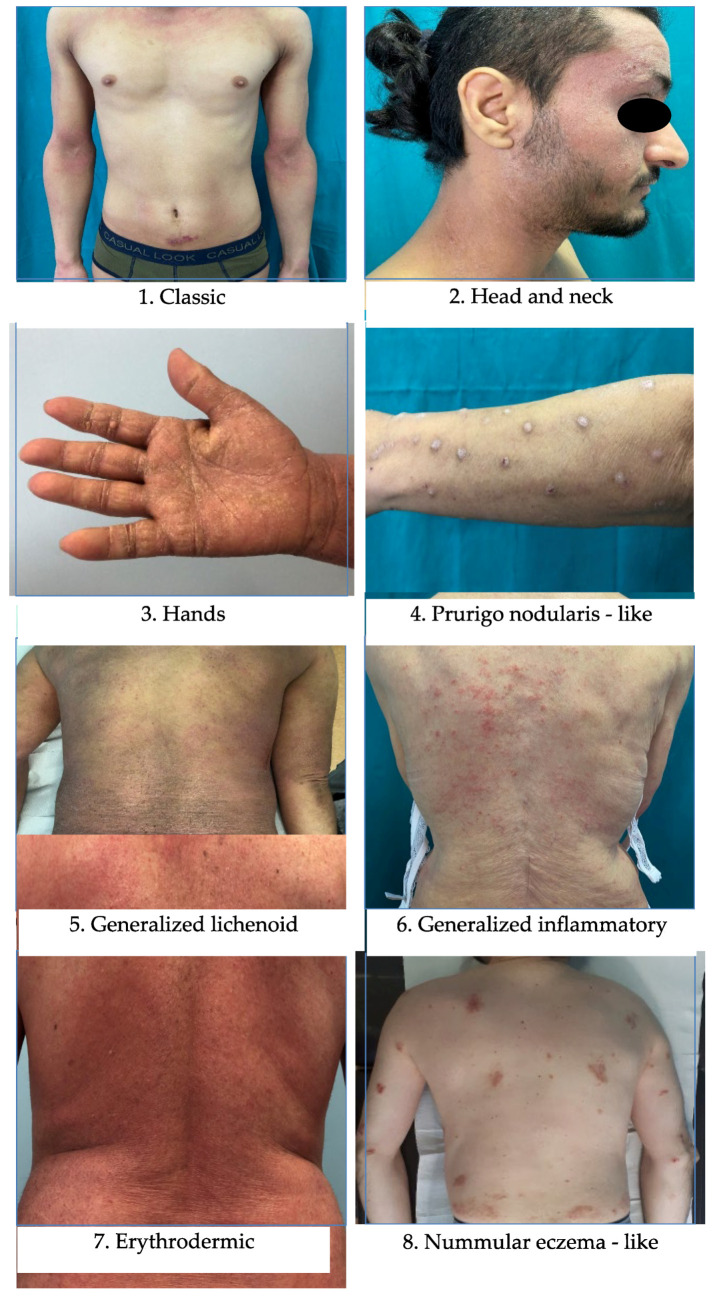
Clinical phenotypes of atopic dermatitis [27]. Pictures kindly provided by S. M. Ferrucci.

Patients’ demographic and clinical baseline characteristics collected are summarized in Table 1: demographics, clinical history of the AD (age at AD diagnosis, age at dupilumab initiation, and duration of the disease), familiarity for atopy, allergic comorbidities (i.e., allergic rhinitis, allergic asthma, and conjunctivitis), concomitant and previous treatments, AD onset pattern, and AD phenotype (Figure 1).

Additionally, laboratory data, particularly total serum IgE levels and peripheral blood eosinophil count, were examined in order to attain a more comprehensive understanding of the correlation between clinical parameters and treatment response. Total serum IgE levels were determined via immunofluorometric assay and expressed in kU/L.

The safety profile of dupilumab was confirmed by recording and monitoring the incidence and severity of adverse events, as well as changes in clinical signs.

### 2.2. Statistical Analysis

Data were reported as median (interquartile range, IQR) or counts (percentages). Fisher’s exact test was used to compare proportions. The Wilcoxon signed-rank test was used to compare within-patient variations of continuous variables. Univariate and multivariate logistic regression analyses were performed to identify predictive factors of early response to dupilumab. Variables identified in the univariate analysis as potentially relevant predictors of early clinical response were included in the multivariate analyses. The results were expressed as the OR with the 95% CI. *p* values < 0.05, two-sided, were considered statistically significant. All statistical analyses were performed using SAS statistical software (version 9.4, SAS Institute, Inc., Cary, NC, USA).

## 3. Results

### 3.1. Demographics and Clinical Baseline Characteristics

A total of 492 patients with moderate-to-severe AD were eligible for the study; the cohort included 451 adults and 41 adolescents aged over 12 years.

Analyzing the demographic and clinical characteristics of the study cohort, the majority of participants were male, constituting 56.10%, while females made up 43.90% of the sample. The median age at the time of AD diagnosis was 3 years, with an IQR spanning from 0 to 14 years. Regarding the initiation of dupilumab treatment, the median age was 33 years. The median duration of dermatitis in the cohort was approximately 25 years. This suggests that patients underwent several years of topical and systemic treatments, as indicated by 80.49% having used cyclosporine and 71.54% having taken oral corticosteroids as previous treatments.

Among the participants, the most prevalent comorbidities were rhinitis and asthma, affecting 76.22% of the sample. Conjunctivitis was present in 49.80% of participants at baseline, and 48.17% had both rhinitis and asthma in conjunction with conjunctivitis. A minority, 22.15% of participants, did not exhibit any atopic comorbidities.

Regarding the onset pattern of AD, the majority (76%) experienced early-onset persistent or relapsing symptoms, while 24% had a late onset in the adulthood.

The classic form of AD was the most prevalent, representing 56.71% of the sample, with lichenified/exudative flexural dermatitis alone or associated with portrait dermatitis, head-and-neck dermatitis, or hand eczema. A non-classic form was represented by 43.29% of patients, encompassing generalized lichenoid and inflammatory, prurigo nodularis, nummular, and erythrodermic variants (Figure 1).

At baseline, participants presented with a median EASI score of 26 (IQR 24–30), a median itch-NRS score of 9 (IQR 8–10), a median sleep-NRS score of 8 (IQR 5–9), and a median DLQI score of 15 (IQR 11–20).

Furthermore, baseline blood data revealed a median total serum IgE level of 1287 kU/L (IQR 286–3906); normal total serum IgE values were defined as <100 kU/L. The median blood eosinophil count was 400 cells/mm^3^ (IQR 250–690), and normal values were defined as <500/mm^3^).

### 3.2. Dupilumab Efficacy

Significant results were discerned within the first four weeks of treatment, with 68.2% of patients achieving a mild level of disease by week 4. Cutaneous lesions, assessed using the EASI score, exhibited substantial amelioration, manifesting a median score of 6 (IQR 6–10), representing a notable improvement from the baseline median of 26. Ninety-three patients (20%) were classified as intermediate responders, as they achieved an EASI score of ≤7 by the time point T4. Additionally, forty-eight patients (10.4%) were categorized as late responders, indicating that they achieved a clinical response in terms of disease severity by either T8 or T12. Only six patients (1.3%) did not achieve disease remission by T12 and were consequently classified as non-responders or partial responders if they were individuals that exhibited clinical improvement that was not sufficient to be considered responders but, at the same time, not significant enough to fit the definition of moderate-to-severe disease, with an EASI score ranging from 7 to 16.

Furthermore, at the T1 assessment, dupilumab revealed pronounced effects, particularly concerning sleep quality and pruritus. Relative to baseline, there was a median percentage reduction −55.6% in the itch-NRS and −71.4% in the sleep-NRS. At T4, sleep-NRS further decreased, reaching a reduction of −100%. These findings underscore the paramount importance of achieving a reduction in both pruritus and clinical severity of cutaneous lesions for patients with moderate-to-severe AD to attain restful sleep, a vital contributor to overall quality of life. Indeed, through the evaluation of the DLQI questionnaire score, it was ascertained that 59.7% of our cohort had reached a DLQI value of ≤10 at T1, signifying a median percentage reduction of −64.1% compared to the baseline.

With regard to the analysis of laboratory parameters, the eosinophil count had a slightly significant increase at T4 (median 460 cells/mm^3^) followed by a decrease at T8 (median 400 cells/mm^3^) and T12 (median 370 cells/mm^3^) in agreement with dupilumab registration and real-life studies [28,29]. Eosinophil count and total serum IgE levels were measured at all clinical assessments in 252 and 194 patients, respectively. A significant and progressive decrease of total IgE levels was observed with a drop from a baseline median of 1382 kU/L (IQR 340–4727 kU/L) to 300 kU/L (IQR 100–908 kU/L) at T12 (median decrease 946 kU/L, *p* < 0.001).

### 3.3. Predictive Factors of Response at 4 Weeks

The multivariate logistic regression analysis did not reveal any predictive value for early clinical response concerning baseline eosinophil count, total IgE levels, and various demographic and clinical parameters, including sex, age at AD diagnosis, AD onset pattern, treatment at baseline, and the use of topical corticosteroids. Notably, as delineated in Table 2, certain variables initially identified as potential predictors of early clinical response to dupilumab in the univariate analysis, specifically eosinophilia and the application of topical steroids at T0, exhibited a loss of statistical significance in the multivariate analysis. In contrast, classic phenotype (Odds Ratio [OR] = 6.92, 95% CI 2.04–23.48) and generalized lichenoid and inflammatory phenotype (OR = 4.22, 95% CI 1.22–14.66) exhibited the predictive value for early response to dupilumab when compared to the nummular eczema phenotype. Additionally, an EASI score ≤ 24 or a score ranging between 24 and 29 at baseline was associated with increased odds of early response compared to a baseline EASI score ≥ 29 (OR = 3.13, 95% CI 1.81–5.41 and OR = 1.79, 95% CI 1.05–3.07, respectively) (Table 2).

### 3.4. Safety and Discontinuation

The safety profile of dupilumab was assessed by analyzing the incidence of the most common adverse effects over the 52-week follow-up period. During this period, 16% of patients developed blepharoconjunctivitis [30,31], and 11.8% experienced facial redness dermatitis [29,32].

Our statistical analyses yielded significant insights when investigating the development of conjunctivitis as an adverse event, taking into account patients’ prior histories of conjunctivitis. Among the 247 patients who had no conjunctivitis at baseline (T0), 25 (10.12%) subsequently developed this adverse event, while among the 245 patients who had a history of conjunctivitis, 56 (22.86%) experienced it after initiating dupilumab treatment. Notably, an association was identified between the presence of atopic conjunctivitis at T0 and the development of blepharoconjunctivitis during dupilumab treatment (*p* < 0.001).

Shifting our focus to the side effect of facial redness dermatitis, a significant distinction emerged when comparing patients with and without facial involvement at T0 (*p* = 0.0081). Remarkably, none of the 53 patients with a clear facial complexion at baseline experienced facial redness as an adverse effect. However, among the 439 patients with facial skin involvement at T0, 52 (11.85%) developed facial redness dermatitis.

Discontinuation due to mild adverse events within one year of treatment occurred in 12 patients (2.4%). Causes were attributed to ineffectiveness (four patients), facial redness dermatitis (three patients), blepharoconjunctivitis (four patients), and psoriasis (three patients). Two patients discontinued treatment due to concurrent facial redness dermatitis and conjunctivitis. The suspension was temporary for six patients due to pregnancy, medically assisted reproduction, SARS-CoV-2 illness, or other unspecified reasons.

## 4. Discussion

Our research findings corroborate the efficacy and safety of dupilumab as a treatment modality for individuals affected by moderate-to-severe AD as proven in clinical trials [11,13].

The classification of patients according to their speed of response to dupilumab, reaching an EASI score ≤ 7, facilitated the investigation of potential drug response determinants. This analysis emphasized the cohort of patients who achieved clinical remission of AD and particularly all the patients that reached a mild severity of AD within a four-week period, referred to as the early responders.

Notably, within the initial four weeks of treatment, a mild level of disease was obtained by 68% of patients treated with dupilumab, resulting in a transition toward a less severe disease state. At the same timepoint, a significant reduction in score pertaining to pruritus (assessed via itch-NRS), sleep disturbances (evaluated using sleep-NRS), and the adverse impact on the quality of life (measured with the DLQI) was achieved. Consequently, dupilumab has demonstrated a pivotal role in the management of patients affected by severe AD, thereby aligning with the World Health Organization’s definition of health as “a state of complete physical, mental, and social well-being” [33].

Moreover, it is noteworthy that our study has affirmed the favorable safety profile of dupilumab, thereby corroborating the evidence from clinical trials [11,12,13] and real-life studies [34,35,36]. Among the most frequently encountered adverse events within our cohort were blepharoconjunctivitis and facial redness dermatitis, with a very low rate of treatment discontinuation. Significantly, these adverse events exhibited associations with specific baseline characteristics. Blepharoconjunctivitis displayed a heightened likelihood of manifesting in patients with a history of conjunctivitis, while facial redness dermatitis predominantly afflicted individuals with facial involvement of AD at baseline.

Regardless of variations in total serum IgE levels and eosinophil counts at baseline, the therapeutic effectiveness of dupilumab remained consistently evident across all patients, thereby highlighting the independence of dupilumab’s efficacy from these blood parameters.

Our study identified specific AD phenotypes within the patient population that served as predictive indicators of an early response to dupilumab therapy. The classic and generalized lichenoid and inflammatory phenotypes exhibited elevated odds of an early response compared to the nummular eczema phenotype. Additionally, patients presenting with a baseline EASI score of ≤24 (adolescents) and those with an EASI score ranging between 24 and 29 were more inclined to display a rapid response to dupilumab.

Limitations of our study include its retrospective nature, which introduces the potential for inherent biases associated with such designs. Although the sample size is noteworthy, larger cohorts would enhance the study’s statistical power and the generalizability of its findings. However, the study’s strengths are rooted in its capacity to furnish real-world clinical practice data and its inclusion of a diverse patient population, encompassing both adults and adolescents.

## 5. Conclusions

The scientific literature currently lacks comprehensive studies that investigate the predictive factors influencing the clinical response to dupilumab in real-world scenarios, thus rendering our study of considerable significance. Our findings suggest that the judicious and timely administration of dupilumab can efficiently and expeditiously ameliorate the disease, offering promising prospects for the personalized management of AD (Figure 1).

## Figures and Tables

**Table 1:** Demographics and clinical baseline characteristics of patients included (N = 492).

Sex	
Men, n (%)	276 (56.10%)
Women, n (%)	216 (43.90%)
Age at diagnosis of AD, years, median (IQR)	3 (0–14)
Age at dupilumab initiation, years, median (IQR)	33 (22–50)
Adults (n)	451
Adolescents (n)	41
Duration of AD, years, median (IQR)	25.5 (17–39)
Familiarity for atopy, n (%) Missing data	213 (55% of 384) 108
Atopic comorbidities, n (%)	
Rhinitis and asthma	375 (76.22%)
Conjunctivitis	245 (49.80%)
Rhinitis and asthma and conjunctivitis	237 (48.17%)
None	109 (22.15%)
Atopic sensitization, n (%)	399 (81%)
AD onset pattern:	
Early-onset persistent or relapsing, n (%)	373 (76%)
Late-onset ‡, n (%)	119 (24%)
AD type according to IgE:	
Intrinsic, n (%)	42 (9.9%)
Extrinsic, n (%)	384 (90.1%)
AD phenotype:	
Classic *	279 (56.71%)
Non-classic **	213 (43.29%)
Generalized lichenoid and inflammatory	138
Prurigo nodularis	41
Nummular eczema	19
Erythrodermic	15
Previous treatments, n (%):	
Cyclosporine	396 (80.49%)
Oral corticosteroids	352 (71.54%)
Others #	190 (38.62%)
Treatment at T0, n (%):	
Cyclosporine	53 (11.23%)
Topical steroids	237 (50.21%)
Cyclosporine and topical steroids	34 (7.20%)
None	148 (31.36%)
Baseline EASI, median (IQR)	26 (24–30)
Baseline itch-NRS, median (IQR)	9 (8–10)
Baseline sleep-NRS, median (IQR)	8 (5–9)
Baseline DLQI, median (IQR)	15 (11–20)
Baseline total serum IgE (kU/L), median (IQR)	1287 (286–3906)
Missing data	96
Baseline blood eosinophil count (cells/mm^3^), median (IQR)	400 (250–690)
Missing data	65

AD, atopic dermatitis; DLQI, dermatology life quality index; EASI, eczema area and severity index; NRS, numerical rating scale; IQR, interquartile range. ‡ Late-onset AD (≥18 years); * Classic phenotype: lichenified/exudative flexural dermatitis alone or associated with head-and-neck, portrait dermatitis, or hand eczema; ** Non-classic phenotype: generalized lichenoid and inflammatory AD, prurigo nodularis, nummular eczema, erythrodermic. # Methotrexate, Azathioprine, Toctine, or phototherapy.

**Table 2:** Univariate and multivariate logistic regression models predicting early response at 4 weeks to dupilumab.

		Univariate		Multivariate	
Variables		OR (95% CI)	*p* Value	OR (95% CI)	*p* Value
Sex	M	1 *	0.0795		-
	F	1.40 (0.96–2.04)			
Age at AD diagnosis (years)	≥65 *	1 *	0.1852		-
	46–65	1.30 (0.64–2.62)			
	19–45	1.31 (0.69–2.50)			
	≤18	2.44 (1.05–5.70)			
AD onset pattern	Late-onset *	1 *	0.6543		-
	Early-onset relapsing	1.27 (0.76–2.14)			
	Early-onset persistent	1.13 (0.72–1.78)			
AD phenotype	Nummular eczema *	1 *	0.0001	1 *	0.0009
	Classic	4.03 (1.56–10.41)		6.92 (2.04–23.48)	
	Generalized lichenoid and inflammatory	1.64 (0.62–4.32)		4.22 (1.22–14.66)	
	Prurigo nodularis	1.19 (0.40–3.56)		2.28 (0.57–9.12)	
	Erythrodermic	0.92 (0.23–3.63)		1.97 (0.38–10.22)	
Treatment at baseline	Cyclosporine + topical steroids *	1 *	0.0606		-
	None	1.58 (0.71–3.49)			
	Cyclosporine only	1.06 (0.43–2.62)			
	Topical steroids only	0.84 (0.40–1.79)			
Use of topical corticosteroids	Yes *	1 *	0.0140		NS
	No	1.64 (1.10–2.43)			
Baseline blood eosinophil count (cells/mm^3^)	≥500 *	1 *	0.0102		NS
	<500	1.68 (1.13–2.51)			
Baseline total serum IgE (kU/L)	≥5001 *	1 *	0.5919		-
	<100	1.27 (0.60–2.69)			
EASI at baseline	≥29 *	1 *	<0.0001	1 *	0.0002
	≤24	2.99 (1.90–4.70)		3.13 (1.81–5.41)	
	24–29	1.83 (1.15–2.91)		1.79 (1.05–3.07)	

AD, atopic dermatitis; EASI, Eczema Area and Severity Index; Late-onset AD (≥18 years); Classic phenotype: lichenified/exudative flexural dermatitis alone or associated with head-and-neck, portrait dermatitis, or hand eczema; OR, Odds Ratio; CI, confidence interval; * Reference category. Multivariate model: all the variables statistically significant at univariate analyses were included. A stepwise selection approach was used to identify variables to be included in the final multivariate model. NS: variables statistically significant at univariate analyses, included in the multivariate model, but excluded from the final multivariate model after stepwise procedure.

## Data Availability

Data are available from the corresponding author upon reasonable request.
